# Adaptive Integration of the Compressed Algorithm of CS and NPC for the ECG Signal Compressed Algorithm in VLSI Implementation

**DOI:** 10.3390/s17102288

**Published:** 2017-10-09

**Authors:** Yun-Hua Tseng, Yuan-Ho Chen, Chih-Wen Lu

**Affiliations:** 1 Department of Engineering and System Science, National Tsing Hua University, Hsinchu 300, Taiwan; s101011806@m101.nthu.edu.tw; 2 Department of Electronic Engineering, Chang Gung University, Taoyuan 333, Taiwan; 3 Department of Radiation Oncology, Chang Gung Memorial Hospital-Linkou, Taoyuan 333, Taiwan

**Keywords:** compressed sensing, electrocardiogram, near-precise compressed algorithm, adaptive integrating compressed algorithm, signal-to-noise ratio, compressed ratio

## Abstract

Compressed sensing (CS) is a promising approach to the compression and reconstruction of electrocardiogram (ECG) signals. It has been shown that following reconstruction, most of the changes between the original and reconstructed signals are distributed in the Q, R, and S waves (QRS) region. Furthermore, any increase in the compression ratio tends to increase the magnitude of the change. This paper presents a novel approach integrating the near-precise compressed (NPC) and CS algorithms. The simulation results presented notable improvements in signal-to-noise ratio (SNR) and compression ratio (CR). The efficacy of this approach was verified by fabricating a highly efficient low-cost chip using the Taiwan Semiconductor Manufacturing Company’s (TSMC) 0.18-μm Complementary Metal-Oxide-Semiconductor (CMOS) technology. The proposed core has an operating frequency of 60 MHz and gate counts of 2.69 K.

## 1. Introduction

According to the World Health Organization (WHO), annual mortality from cardiovascular disease is expected to increase from 17.5 million in 2012 to 22.2 million in 2030, and annual cancer deaths are expected to climb from 8.2 million to 12.6 million during the same period [1]. In order to prevent cardiovascular disease in advance, personal heart monitors have been developed to safeguard a wide variety of human activities. The wireless body sensor network (WBSN) is a special class of wireless sensor network (WSN) comprising various types of miniature biosensors, which are worn or implanted for the continuous monitoring of biomedical signals, such as those from an electrocardiogram (ECG). These devices require an algorithm for the compression and subsequent storage of data. The signal-to-noise ratio (SNR) and compression ratio (CR) are important parameters when dealing with these types of algorithms. Some of the compression algorithms are bit-accurate (high SNR but low CR) whereas others are lossy (high CR but low SNR). Advances in semiconductor technology have made it possible to use integrated circuits (ICs) for remote monitoring for over 40 years where flexibility and portability are important. Some hardware design issues like power consumption, hardware cost, and recovery performance are crucial to the effectiveness of portable devices, and researchers have proposed algorithms to specifically deal with these issues. 

In lossless ECG compression algorithm research, a low-complexity lossless compression algorithm based on an adaptive trending prediction and two-stage Huffman coding was proposed in [2]. The architecture proposed in [2] includes a two-stage Huffman table that may lead to an increase in the hardware cost. In order to reduce the hardware cost, a lossless compression algorithm based on fuzzy decision control and hybrid entropy coding was developed by same author and a two-stage Huffman table technique that can be separated into two small tables to reduce hardware costs efficiently was proposed in [3]. According to the synthesis result in [3], the number of gate counts and area can be reduced efficiently compared with the architecture in [2]. Some low-hardware costs and low-power consumption designs are also developed in another work. One lossless algorithm using fuzzy-based PSO prediction and Huffman region entropy coding was described in [4], and the power consumption and hardware cost was reduced more effectively with system-on-chip (SoC) in wireless ECG sensors devices proposed in [5] than in previous lossless research [2,3]. 

Although the previous lossless ECG compression researches provides good performance but the CR is low, and it is necessary to develop a compression algorithm to balance CR and recovery performance. In recent years, some novel compression algorithms have been developed based on compressed sensing (CS) technology to compress sparse signals, and then reconstruct them by exploiting their sparsity [6,7,8,9]. This type of compression algorithm uses a simple linear transform (sensing matrix) to compress data, and hardware implementation can be achieved. Some researchers have developed CS compression algorithms to improve hardware issues like cost, reconstruction performance and power consumption. For example, minimal mutual-coherence pursuit (MMCP) proposed by [6] based on the CS algorithm was proposed for the construction of a sparse binary matrix (SBM) capable of encoding ECG records with high sensitivity and ultra-low energy consumption. In [7], researchers proposed using the CS algorithm for the monitoring of ECG signals with prior probability of sparsity in the wavelet domain using a variable orthogonal multi-matching pursuit algorithm to reduce hardware costs and energy consumption. In [8], an adaptive dictionary reconstruction scheme was developed to improve CS performance when dealing with electrocardiogram signals.

**Figure 1:**
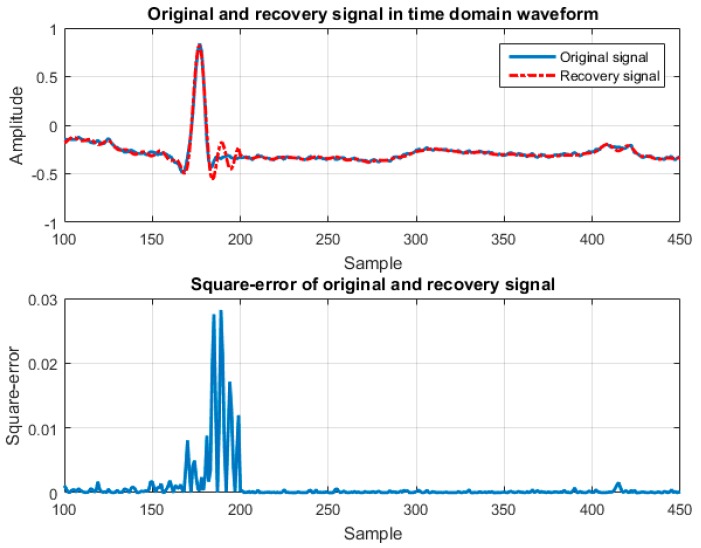
Square error of signal window when using the compressed sensing (CS) algorithm proposed in [9].

As mentioned previously, the reconstruction performance of lossless compression algorithms is superior and hardware implementation with high CR can be achieved by the CS algorithm. The algorithm proposed in this paper is referred to as the adaptive CS/ near-precise compressed (NPC) compression algorithm, which is designed integrating the advantages of CS and NPC to achieve high CR and high SNR. Figure 1 presents the waveform of the original signal and the signal that was reconstructed using CS [9] and the square-error of these two signals. Square error is convenient but may not be the ideal measure to assess the real-world performance of a reconstruction algorithm. For instance, in a remote ECG monitor, the intended use might not reflect an accurate measure of the QRS reconstruction, but may detect arrhythmia. In order to detect cardiac arrhythmias, the QRS complex needs to be detected and the timing of the R-peak should be highly accurate between the original and reconstructed signal—a high square error may not be a problem in real life and depends on the specific purpose of other measures. As shown in Figure 1, most of the changes between the original and reconstructed signals are distributed in the QRS region. Furthermore, recovery performance decreases following any increase in the compression ratio. The high square-error region around the QRS signal was compressed using the NPC algorithm, whereas the other regions underwent compression using the CS algorithm. First, the R–R interval is divided into the data length of the CS-compressed region (Ncs) and the data length of the NPC compressed region (Nnpc). The input data X(t) is then divided according to Ncs and Nnpc and fed into the CS or NPC algorithm to undergo data compression. The performance of the proposed scheme was evaluated using the Massachusetts Institute of Technology and Boston's Beth Israel Hospital (MIT-BIH) Arrhythmia Database. The simulation results demonstrate that the SNR and CR of the proposed scheme are balanced and the hardware implementation can be easily achieved. Furthermore, the proposed algorithm is also implemented in a single chip using the Taiwan Semiconductor Manufacturing Company’s (TSMC) 0.18-μm Complementary Metal-Oxide-Semiconductor (CMOS) technology. The proposed chip achieves a low-cost design with only 2.69-K gate counts. The proposed design is also implemented into the Field-Programmable Gate Array (FPGA) platform to demonstrate the compression performance. The results show low hardware resource is utilized when the proposed design is implemented into the Xilinx Kintex-7 FPGA.

The remainder of this paper is organized as follows. In Section 2, we present the theory underlying the proposed algorithm. A comparison of synthesis results is presented in Section 3, and a discussion is presented in Section 4. In Section 5, we describe the implementation of the proposed chip. Conclusions are drawn in Section 6.

## 2. Proposed Architecture

**Figure 2:**
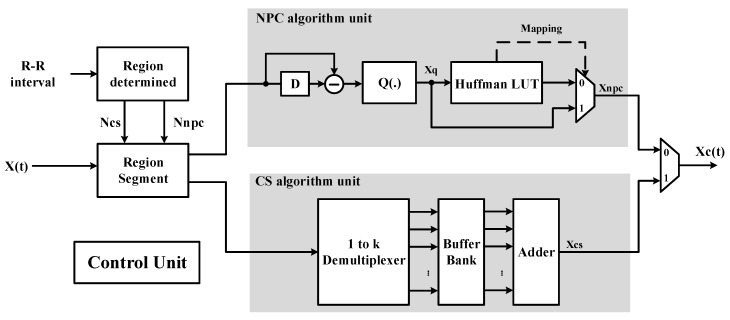
Proposed architecture. NPC: near-precise compressed.

Figure 2 presents the architecture of the adaptive CS/NPC compression algorithm. The architecture of the compressing unit includes two parts: an NPC algorithm and a CS algorithm. The high change region of the original and recovery ECG signal is compressed by the NPC algorithm and the other region is compressed by a CS algorithm. First, the R–R interval is divided into Ncs and Nnpc and the two values are data lengths of the CS region and NPC region, respectively. The input data X(t) is divided into two-part data according to Ncs and Nnpc, and this information is respectively fed into CS and NPC algorithm units to compress the data. The control unit controls multiplexers in architecture to switch signals. A description of the block in proposed architecture is in the following subsection. 

### 2.1. Compressed Sensing

CS relies on the sparsity of underlying sampled signals for compression and reconstruction. Let $x\in {\mathbb{R}}^{N}$ be a K-sparse or compressible signal with respect to the basic $\Psi =\left[\psi_{1}\;\psi_{2}\;\cdots \;\psi_{N}\right]$, as long as the transform $\alpha \in {\mathbb{R}}^{N}$ contains no more than K nonzeros, as follows:

(1) $$x\approx \overset{N}{\underset{k=1}{\sum }}a_{k}\psi_{k}=\Psi \alpha$$

where $\alpha =\;{\left[\alpha_{1}{\;\alpha }_{2}\;\cdots {\;\alpha }_{N}\right]}^{T}$ with K nonzero elements as K≪N is a K-sparse vector. CS compresses the K-sparse signal x by multiplying the measurement or sensing matrix $\Phi \in {\mathbb{R}}^{M\times N}$, where K<M<N.

The resulting y is called measurement vector and is expressed as follows:

(2) y=Φx=Ωα

where $\;\Omega \in {\mathbb{R}}^{M\times N}=\Phi \Psi$. Signal  α can be reconstructed from measurement M as long as the sensing matrix obeys the property of mutual coherence [10]. The sparse vector $\hat{\alpha }$ takes the form from $\ell_{1}$-minimization, as follows:

(3) $$min{\left||\hat{\alpha }|\right|}_{1}\;\mathrm{subject}\;\mathrm{to}\;y=\Phi \Psi \hat{\alpha }=\Omega \hat{\alpha }.$$

Once the sparse vector $\hat{\alpha }$ has been obtained, the reconstruction original vector $\hat{x}$ can be obtained as follows:

(4) $$\hat{x}=\Psi \hat{\alpha }.$$

Some algorithms, such as orthogonal matching pursuit (OMP) [11], extend the orthogonal matching pursuit [12], iterative hard threshold (IHT) [13], and gradient pursuit (GP) [14] to find an appropriate solution for $\hat{\alpha }$ in (2) for CS recovery.

#### 2.1.1. Measurement Matrix 

The measurement matrix Φ and sparsity base Ψ are incoherent in (3). The coherent factor β between measurement matrix Φ and sparsity base Ψ is expressed as follows:

(5) $$\beta \left(\Phi ,\Psi \right)=\sqrt{N}\underset{i,j}{\max}\frac{\left|\langle \Phi_{i},\Psi_{j}\rangle \right|}{{\left||\Phi_{i}|\right|}_{2}{\left||\Psi_{j}|\right|}_{2}}\;$$

where $\Phi_{i\in \left\{1,2,\ldots ,M\right\}}$ and $\Psi_{j\in \left\{1,2,\ldots ,N\right\}}$ respectively represent the row of matrix Φ and column of matrix Ψ and compressive sampling is concerned mainly with areas of low coherence [10]. In general for CS algorithm applications, random matrices such as Gaussian or Bernoulli matrices are suitable for measurement matrix Φ. To further reduce hardware costs, we adopted the following binary block diagonal measurement matrix proposed in [9]:

(6) $$\Phi =\left[\begin{array}{cccc} \overset{z}{\overbrace{\left[1\ldots 1\right]}} & 0 & \cdots & 0\\ 0 & \overset{z}{\overbrace{\left[1\ldots 1\right]}} & \cdots & \vdots \\ \vdots & \vdots & \ddots & 0\\ 0 & 0 & \cdots & \overset{z}{\overbrace{\left[1\ldots 1\right]}} \end{array}\right]$$

where the diagonal element *z* = *N*/*M*; and *M* and *N* represent the number of rows and columns respectively. The measurement matrix in (6) is easy to implement because it requires only a few adders and the area of the adder is smaller than that of the multiplier in very large-scale integration (VLSI). Thus, the proposed architecture makes it possible to reduce power consumption and hardware cost of the microcompressor.

#### 2.1.2. Discrete Cosine Transform (DCT) 

The nonzero element K of the sparsity base is an important factor in (1). A lower k-factor enables better recovery performance. The DCT transform is a conventional algorithm used to reduce the *k* value. It is widely used in image and video compression [15] and it can be bit-accurate as well to compress ECG signals in [16]. The transform matrix of DCT is expressed as follows:

(7) $$\Psi_{u,v}=k_{u}\cos\left(u\left(1+2v\right)\frac{\pi }{2N}\right)$$

where u∈{0,1,…,N−1} and v∈{0,1,…,N−1} represent the number of row and column matrices, respectively. The coefficient $k_{u}=1/\sqrt{N}$ for u=0 and $k_{u}=1$ for u≠0. The inverse DCT (IDCT) is a matrix transpose operation because the DCT matrix is orthogonal $\mathrm{IDCT}={\mathrm{DCT}}^{T}$. The advantage of using DCT in CS is the fact that the output of DCT may produce a high-density collection of zeros in the high-frequency part of the transform block. This means it can be discarded without loss and the predefined threshold can be achieved simply by retaining a few low-frequency signals, as proposed in [10]. The recovery algorithm also utilizes the threshold approach in the DCT domain in order to make the algorithm simpler. For our algorithm, we adopted the recovery algorithm in [10] in order to simplify the signal recovery process.

### 2.2. Near-Precise Compressed Algorithm

Most of the changes from the original signal can be eliminated by using the proposed NPC algorithm, due to the fact that it is highly precise. Furthermore, the NPC algorithm is easily implemented using low-cost hardware. The process of the NPC algorithm is as follows:

1. Differences between adjacent signals are first calculated to reduce the amplitude scale of the signal. Thus, there is a greater probability of the same different result, which makes it possible to increase the compression ratio using methods based on Huffman coding theory.
2. Generally, quantifying differences between adjacent signals requires an infinite number of bits; however, that is impossible to implement in VLSI. Thus, we perform quantization to the eighth decimal place in the NPC algorithm.
3. Huffman coding utilizes symbols that vary in repetition to map bits of different lengths. If the symbol Xq is repeated frequently, then the output data Xnpc will have fewer bits after Huffman mapping. Consequently, symbol Xq (repeating infrequently) is mapped to data of a longer length. To increase the compression ratio and restrict the number of output bits, a multiplexer is switched according to whether the input data are mapped in a Huffman Look-Up Table (LUT). If the input data are mapped in a Huffman LUT, then Mapping = 0 and output data Xnpc are equal to the Huffman LUT mapping results. If the input data are not included in the Huffman LUT, then Mapping = 1 and the output Xnpc is equal to the quantification results.

**Figure 3:**
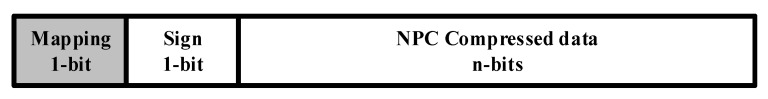
Total output format of NPC algorithm unit.

Figure 3 shows the output format of signal Xnpc. Determining whether data $X_{\mathrm{npc}}$ was mapped in Huffman LUT can be achieved by adding a mapping sign in the first bit of the output format. The second bit is a sign bit indicating whether data Xnpc is positive or negative. The third part of the output presents data bits of Xnpc. The number of bits n ranges from 3≤n≤8 and the bit length is small in cases where items of data are repeated frequently.

The features of the proposed NPC algorithm are summarized in the following:

1. When the symbol Xq is repeated frequently, it is compressed via Huffman coding and the compressed data is restricted to fewer than 8 bits. ECG signals in the QRS region can be compressed with almost no loss using the proposed NPC algorithm.
2. The Huffman LUT can be implemented using low-cost hardware because we map only the portion of the data that appears frequently. This reduces the size of the LUT by 82%.

### 2.3. Adaptive Compression Algorithm Integrating CS and NPC

**Figure 4:**
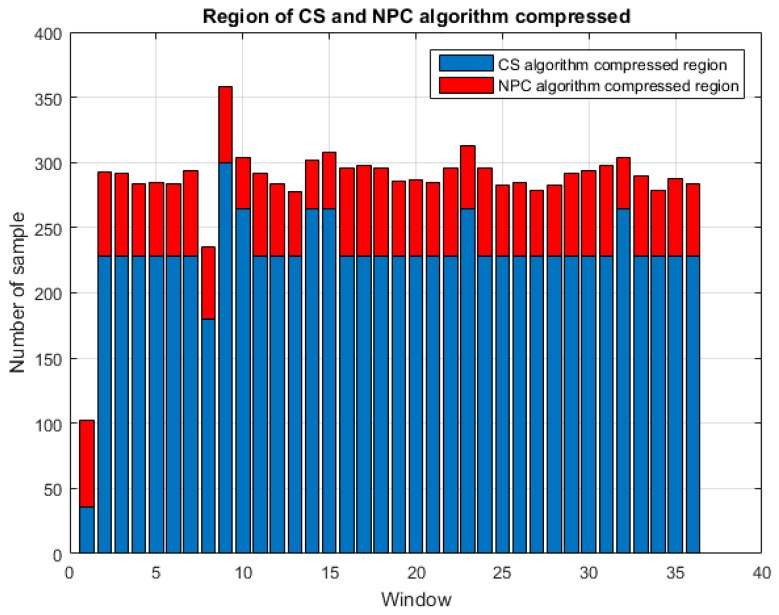
Regions of compression implemented using the CS and NPC algorithms.

A simple measurement matrix and rapid recovery algorithm were proposed in [9]. We sought to improve this architecture with the aim of enhancing recovery performance without giving up on a high compression ratio. The proposed architecture is referred to as the adaptive CS/NPC compression algorithm. The region of high square-error proximal to the QRS signal is compressed using the NPC algorithm, whereas other regions are compressed using the CS algorithm. There are only slight changes in the region of low-square error (compressed by CS), which means the compression ratio can be high without compromising recovery performance. Furthermore, we predefine the compressing region distribution of CS and NPC algorithm compression. Figure 4 illustrates the regions that underwent compression using the CS and NPC algorithms, respectively. Most of the signal window (blue region) undergoes high compression using the CS algorithm. The NPC algorithm is used to apply compression in the region of the QRS signal (red region). The compression ratio of the NPC algorithm is lower than that of the CS algorithm; however, the overall compression ratio is high. Algorithm 1 is the proposed algorithm processing method to clarify the description:
**Algorithm 1. Proposed Adaptive Compressed Algorithm.****Input:** Input data $x\;\epsilon \;{\mathbb{R}}^{w_{r}}$; R–R information $w_{r}$**Output:** Compressed data y**1.**  **Initialization:**
*s = 0*; *i = 0; j = 0*; $w_{r}$ is decomposed to $N_{cs}\;\mathrm{and}\;N_{npc}$, where $w_{r}=N_{cs}+N_{npc}$**2.**  **while (**$s<w_{r}-1$**)****3.**   **(NPC algorithm)****4.**   **while (**$s<N_{npc}-1$**)****5.**    **Adjacent signals difference:** If (*k = 0*), ${\tilde{x}}_{s}=x$, **else**
${\tilde{x}}_{s}=x-{\tilde{x}}_{s-1}$**6.**    **Quantification:**
$x_{q}=Q\left({\tilde{x}}_{s}\right)$**7.**    **Huffman mapping:** If **(**$x_{q}$ maps to Huffman LUT **)**
$x_{npc}=LUT\left(x_{q}\right)$, *mapping = 0***8.**             **else**
$x_{npc}=x_{q}$, *mapping = 1***9.**    **Update NPC output format:**
$y_{npc}=\left[mapping\;x_{npc}\right]$**10.**    **end while****11.**    **(CS algorithm)****12.**    **while** ($N_{npc}<s<N_{cs}-1$)**13.**     if *(*j=0*),*
$x_{j}=x$*,*
**else**
$x_{j}=x+x_{j-1}$**14.**     if *(j =*
$N_{cs}/M-1$)**15.**     $j=0,\;y_{cs,i}\in {\mathbb{R}}^{M}=x_{j}$, i=i+1 where $y_{cs}\in {\left[y_{cs,0},y_{cs,1},\ldots ,y_{cs,N_{cs}/M-1}\right]}^{T}$**16.**     **else****17.**     j=j+1, $x_{j}=x_{j}$**18.**    **end while****19.**   **Index update:**
*s = s + 1***20.**   **Output data:**
$y=\left[y_{npc}\;y_{cs}\right]$**21.**   **end while**

## 3. Simulation Results

**Figure 5:**
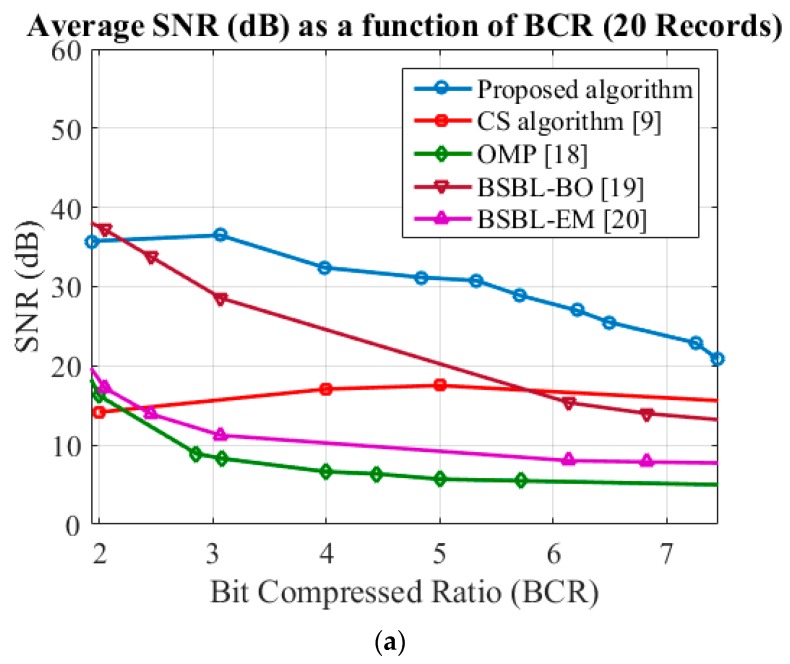

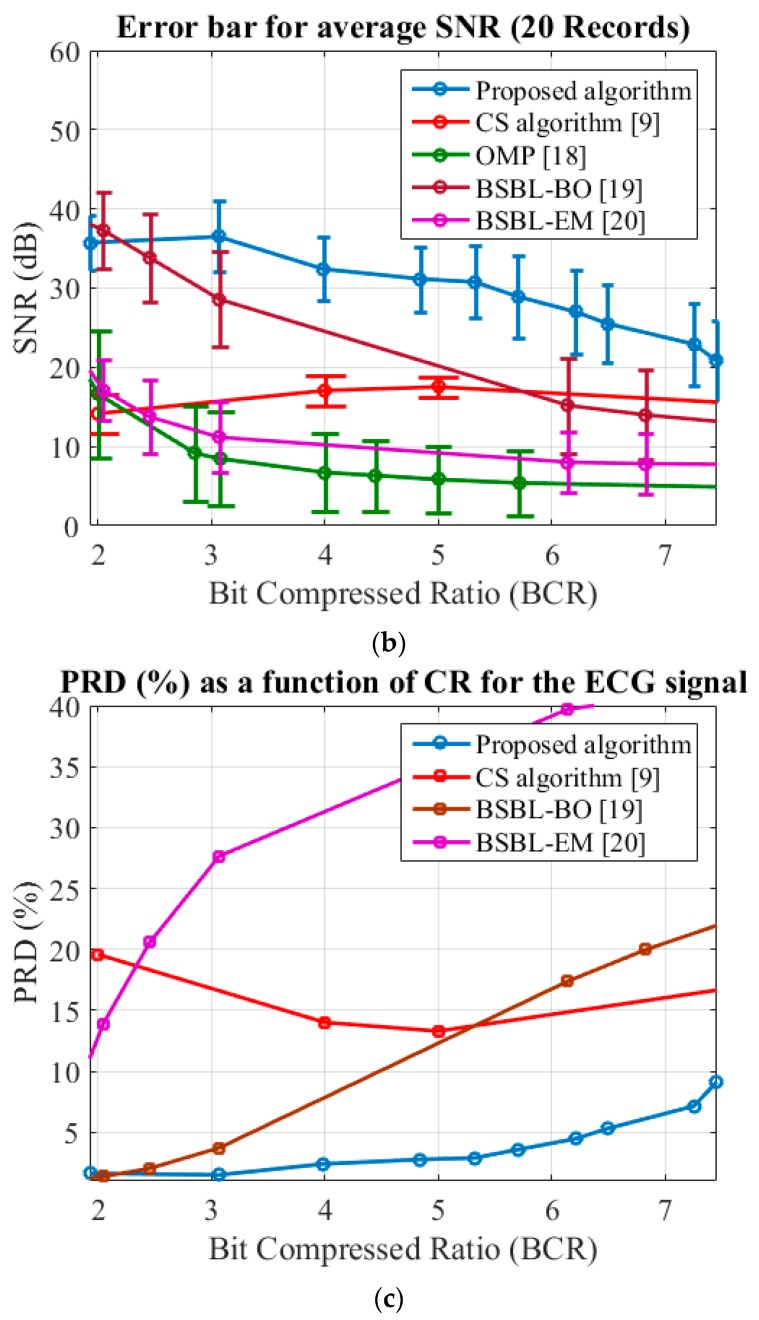
Comparison of various algorithms: (**a**) Signal-to-noise ratio (SNR); (**b**) Error bar for SNR (standard deviation); (**c**) Percent-root-different (PRD).

The performance of the proposed algorithm was evaluated using the MIT-BIH Arrhythmia Database [17], based on the signal-to-noise ratio (SNR) and root-mean-square-difference (PRD), which are defined as follows:

(8) $$\mathrm{SNR}\left(\mathrm{dB}\right)=20\log\left(\frac{{\left||x|\right|}_{2}}{{\left||x-\hat{x}|\right|}_{2}}\right)$$

(9) $$\mathrm{PRD}\left(\%\right)=\left(\frac{{\left||x-\hat{x}|\right|}_{2}}{{\left||x|\right|}_{2}}\right)\times 100$$

where x and $\hat{x}\;$ are the original and recovered signals, respectively. When the SNR is higher and the PRD is lower, the recovery signal is close to the original signal. ECG is a biomedical signal with varying information content for Medical Devices (MDs) and not every acquired sample in the technical sense is of equal importance. Thus, the performance measure (SNR and PRD) are convenient to compare with others but not necessarily ideal to judge on the real-world performance/applicability of their method. The bits-compressed-ratio (BCR) is defined as follows:

(10) $$\mathrm{BCR}=\frac{B_{o}}{B_{c}}$$

where $B_{o}$ is the bit number of the uncompressed data and $B_{c}$ is the bit number of the compressed data. Much more of the data is compressed when parameter BCR is increased. In this section, we simulated and compared some of the architectures proposed in [9,18,19,20] in Figure 5. The architecture proposed in [18] (referred to as the orthogonal matching pursuit, OMP) recovers a signal using a Gaussian random sensing matrix, and the architecture proposed in [19] and [20] are bound-optimization-based block sparse Bayesian learning (BSBL-BO) and expectation-maximum-based block sparse Bayesian learning (BSBL-EM) respectively. The sensing matrix utilizing in BSBL-BO and BSBL-EM are randomly generated sparse binary sensing matrix, with each column consisting of 12 entries of 1 s with random locations, while other entries were all zero [19]. In this study, we sought to improve on the simple measurement matrix and fast recovery algorithm proposed in [9]. We refer to the proposed architecture as the adaptive CS/NPC compression algorithm, which is divided into two compression units: (1) data in the region close to QRS is compressed using the NCP algorithm, and (2) data in other regions is compressed using the CS algorithm. In order to implement the chip, we simulated 10 different CRs of the CS-base algorithm utilizing in our proposed architecture, with values of 2, 4, 8, 10, 12, 15, 20, 25, 30, and 40 respectively and selected the highest quality score (QS) of CR, which was defined as follows:

(11) $$\mathrm{QS}=\frac{\mathrm{CR}}{\mathrm{PRD}}\;$$

Based on the simulation results in Table 1, we selected the highest QS of the CS-base algorithm equaling 12 to implement the chip.

Figure 5 presents the average SNR and PRD values (as a function of CR) when applied to 10 ECG records using various algorithms. Simulations of random 20 records are 100, 101, 111, 112, 113, 114, 115, 116, 117, 118, 119, 121, 103, 122, 123, 124, 212, 220, 230, and 231, respectively. As shown in Figure 5a, the average SNR values obtained using the proposed architecture are better than other algorithms in 20 different records. The error bar for the SNR performance (standard deviation) as shown in Figure 5b presents the deviation of proposed algorithm for the SNR curve is still better than other algorithms in high BCR, and the average PRD is lower than in the CS algorithm proposed by [9] as shown in Figure 5c. According to simulation results, the performance of the proposed architecture was better than that of the other algorithms when CR was increased in various records; however, these benefits were observed only when applied to specific ECG records.

## 4. Discussion

**Figure 6:**
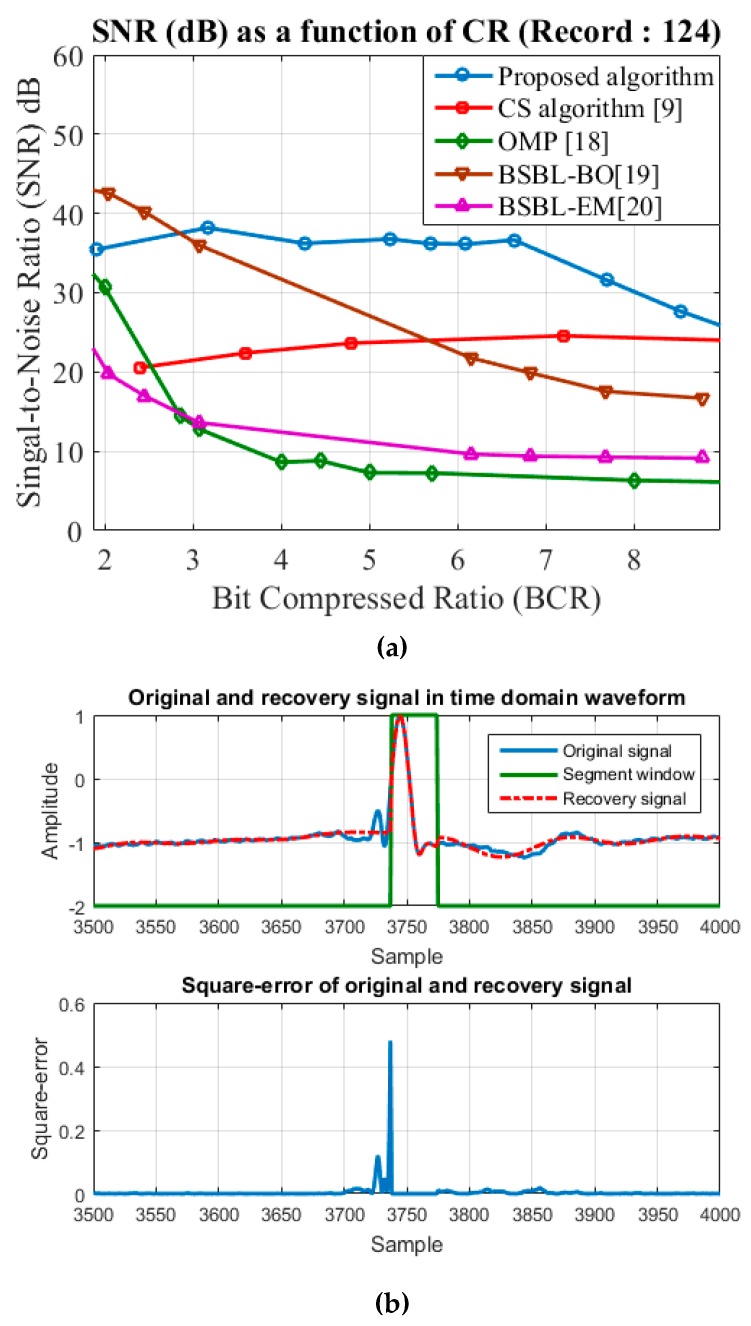
Example simulation using record 124: (**a**) comparison of SNR values obtained using different algorithms; (**b**) squared segment window offset error between the original and the recovery signal.

In this section, we discuss the cause of the changes observed in the recovery process. Figure 6a presents an example of comparison of SNR values obtained using various algorithms when applied to a specific ECG record (No. 124). When the CR was lower, the SNR values obtained using the proposed algorithm were higher than those obtained using CS and OMP. Nonetheless, when the CR was increased, the SNR was close to that obtained using the CS algorithm. These results show that the performance of the proposed algorithm is close to that of the CS algorithm in [9] when CR is high, which was caused by segment window offset error, as shown in the simulation results in Figure 6. The other probable cause of changes is the location of high-beating waveforms. When the high-beating waveforms are located in non-QRS region, the compressed algorithm utilizing the CS algorithm and SNR gives poorer results when using the CS-compressed algorithm in a high- beating waveform in the proposed architecture. These problems cause a drop in the SNR performance in proposed design and thus further improvements are required in the future.

Figure 6b presents the squared segment window offset error between the original and recovery signals in order to identify the source of these changes. Low and high symbols of the segment window are compressing regions of the CS and NPC algorithm, respectively. Figure 6 shows the offset segment window, which leads to compression of parts of the QRS signal by the CS algorithm—the square-error increases when QRS region signals are compressed by the CS algorithm. In order to reduce the probability of segment window offset error, we shift the position of the segment window and increase the compressed region of the NPC algorithm. Although this decreases the CR, it effectively improves the SNR. These results confirm that the proposed architecture achieves high performance.

## 5. Hardware Implementation

### 5.1. Chip Implementation

**Figure 7:**
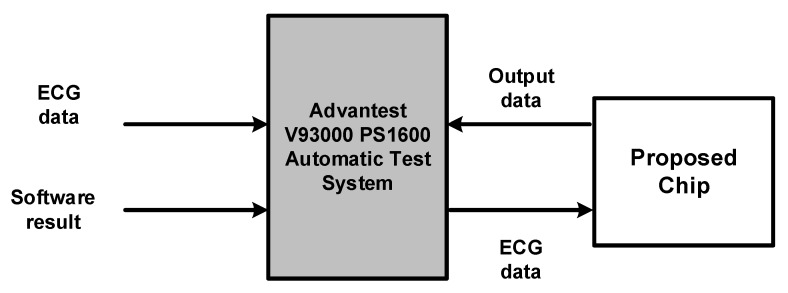
The block diagram of chip testing.

The proposed core was implemented using register-transfer level (RTL) hardware based on the TSMC 0.18-μm standard CMOS technology. Following synthesis using the Synopsys Design Complier, Cadence Encounter digital implementation (EDI) was used for placement and routing (P&R). The chip testing utilizing Advantest V93000 equipment to verify the function is working and its power consumption measurement, as shown in Figure 7. 

**Figure 8:**
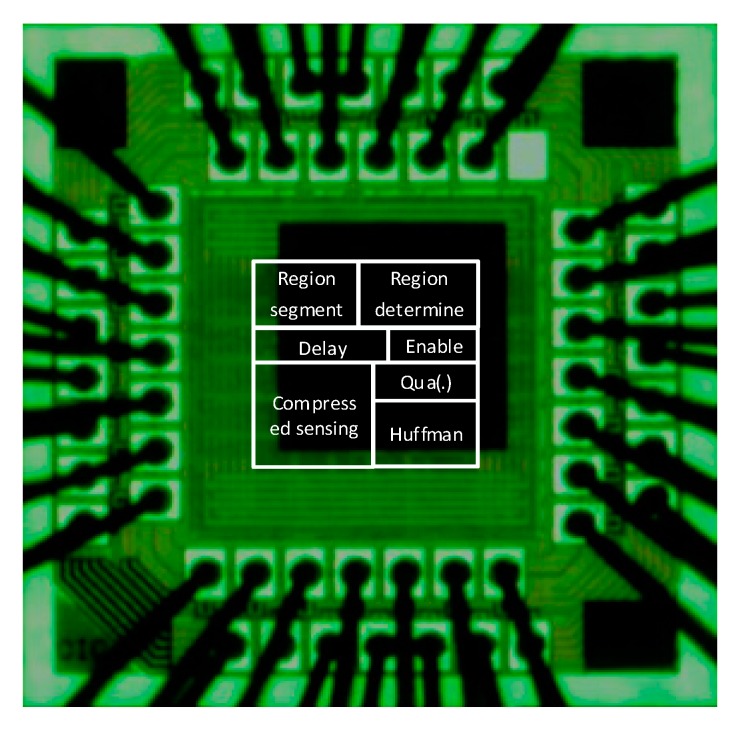
Photomicrograph of the proposed chip.

We load the ECG data from MIT-BIH Arrhythmia Database and fixed-point simulation result from MATLAB to Advantest V93000 equipment, then V93000 generated the ECG data inputting to proposed chip and measured the output data from the chip to verify the function of the proposed chip. We also utilized the V93000 equipment to measure the power consumption and operation frequency of the proposed chip. Table 2 lists the hardware characteristics of proposed chip. The proposed architecture core has a core area of 831 × 827 ${\mu m}^{2}$ and is capable of operation at 60 MHz. The gate count was approximately 2.69 K and the power consumption was 2.1 mW. Figure 8 illustrates the photomicrograph of the proposed chip and Table 2 lists the hardware specifications.

### 5.2. FPGA Implementation

In order to verify the hardware implementation, the proposed architecture was also implemented into FPGA platform in the Kintex-7 FPGA Development Board. The proposed design was synthesized using the Xilinx ISE 14.7 tool, and the Xilinx XC7K325T Kintex-7 FPGA can be operated at clock frequency of 131 MHz. Table 3 presents the characteristics of proposed architecture implemented into Xilinx FPGA. As shown result in Table 3, the proposed compression core utilizes low area resources in the XC7K325T FPGA implementation, and low hardware cost can be achieved by the proposed architecture.

## 6. Conclusions

In this study, we proposed an adaptive algorithm in which CS and NPC are integrated for the compression of ECG signals. The region of high change between the original and recovered signals undergoes compression using a highly precise algorithm referred to as NPC, whereas the other regions are compressed using the CS algorithm. In simulations, the SNR of the proposed algorithm is higher than that obtained using existing algorithms and the PRD is lower than that achieved using the CS algorithm. The proposed core was implemented based on the TSMC 0.18-μm standard CMOS process. The proposed device has a gate count of 2.69 K and power consumption of 2.1 mW. This demonstrates the efficacy of the proposed algorithm in the development of microcompressors with high CR.

## Figures and Tables

**Table 1:** Different QS with varying CRs in the CS-base algorithm utilized in the proposed architecture.

**CR**	2	4	8	10	12	15	20	25	30	40
**QS**	1.50	2.01	1.87	2.07	2.12	1.85	1.55	1.31	0.99	0.85

**Table 2:** Hardware characteristics of the proposed chip.

**Process Technology**	TSMC 0.18 μm CMOS
**Supply Voltage**	1.8 V
**Maximum Frequency**	60 MHz
**Core Area**	831 × 827 ${\mu m}^{2}$
**Power Consumption**	2.1 mW
**Gate Count (K)**	2.69
**Bits-Compressed Ratio**	5.05

**Table 3:** Characteristics of proposed architecture implemented in FPGA.

FPGA Chip	XC7K325T	
	Used	Available
**# of Slices Registers**	126	407,600
**# of Slices LUTs**	428	203,800
**# of Fully-Used LUT-FF Pairs**	102	452
**Clock Frequency**	131 MHz	

## References

[1] WHO (2015). Global Status Report on Noncommunicable Disease 2014.

[2] Chen S.L., Wang J.G. (2013). VLSI implementation of low-power cost-efficient lossless ECG encoder design for wireless healthcare monitoring application. Electron. Lett..

[3] Chen S.L., Lo K.A., Lin T.L. (2013). Efficient fuzzy-controlled and hybrid entropy coding strategy lossless ECG encoder VLSI design for wireless body sensor networks. Electron. Lett..

[4] Chen S.L., Tuan M.C., Chi T.K., Lin T.L. (2015). VLSI architecture of lossless ECG compression design based on fuzzy decision and optimization method for wearable devices. Electron. Lett..

[5] Deepu C.J., Zhang X., Liew W.S., Wong D.L.T., Lian Y. (2014). An ECG-on-Chip with 535 nW/Channel Integrated Lossless Data Compressor for Wireless Sensors. IEEE J. Solid-State Circuits.

[6] Zhang J., Gu Z., Yu Z., Li Y. (2015). Energy-efficient ECG compression on wireless biosensors via minimal coherence sensing and weighted l1 minimization reconstruction. IEEE J. Biomed. Health Inform..

[7] Cheng Y.C., Tsai P.Y., Huang M.H. (2016). Matrix-Inversion-Free Compressed Sensing with Variable Orthogonal Multi-Matching Pursuit Based on Prior Information for ECG Signals. IEEE Tran. Biomed. Circuit Syst..

[8] Craven D., McGinley B., Kilmartin L., Glavin M., Jones E. (2017). Adaptive Dictionary Reconstruction for Compressed Sensing of ECG Signals. IEEE J. Biomed. Health Inform..

[9] Ravelomanantsoa A., Rabah H., Rouane A. (2015). Compressed sensing: A simple deterministic measurement matrix and a fast recovery algorithm. IEEE Trans. Instrum. Meas..

[10] Candès E.J., Wakin M.B. (2008). An introduction to Compressive Sampling. IEEE Signal Process. Mag..

[11] Wang J., Kwon S., Shim B. (2012). Generalized orthogonal matching pursuit. IEEE Trans. Signal Process..

[12] Sahoo S., Makur A. (2015). Signal recovery from random measurements via extended orthogonal matching pursuit. IEEE Signal Process. Soc..

[13] Blumensath T., Davies M.E. (2010). Normalized iterative hard thresholding: Guaranteed stability and performance. IEEE J. Sel. Top. Signal Process..

[14] Blumensath T., Davies M.E. (2009). Stagewise weak gradient pursuits. IEEE Trans. Signal Process..

[15] Chen Y.H., Chang T.Y., Li C.Y. (2012). A high performance video where transform engine by using space-time scheduling strategy. IEEE Trans. VLSI Syst..

[16] Lee H., Buckley K.M. (1999). ECG data compression using cut and align beats approach and 2-D transforms. IEEE Trans. Biomed. Eng..

[17] Moody G.B., Mark R.G. (2001). The impact of the MIT-BIH arrhythmia database. IEEE Eng. Med. Biol. Mag..

[18] Tropp J.A., Gilbert A.C. (2007). Signal recovery from random measurements via orthogonal matching pursuit. IEEE Trans. Inf. Theory.

[19] Zhang Z., Jung T.-P., Makeig S., Rao B. (2013). Compressed sensing for energy-efficient wireless telemonitoring of noninvasive fetal ECG via block sparse bayesian learning. IEEE Trans. Biomed. Eng..

[20] Zhang Z., Wei S., Wei D., Lin L., Liu F., Liu C. Comparison of Four Recovery Algorithms Used in Compressed Sensing for ECG Signal Processing. Proceedings of the Computing in Cardiology Conference (CinC).

